# Analysis of VISTA expression and function in renal cell carcinoma highlights VISTA as a potential target for immunotherapy

**DOI:** 10.1007/s13238-019-0642-z

**Published:** 2019-06-24

**Authors:** Shanjuan Hong, Qing Yuan, Haizhui Xia, Genzhen Zhu, Yu Feng, Qiang Wang, Zhiyin Zhang, Wei He, Jian Lu, Chen Dong, Ling Ni

**Affiliations:** 1 Institute for Immunology and School of Medicine, Tsinghua University, Medical Research Building, Beijing, 100084 China; 2 Division of Urology, The 8th Medical Center of Chinese PLA General Hospital, Beijing, 100091 China; 3 Department of Urology, Peking University Third Hospital, Beijing, 100191 China; 4 Department of Urology, School of Clinical Medicine, Beijing Tsinghua Changgung Hospital, Tsinghua University, Beijing, 102218 China; 5 Beijing Key Lab for Immunological Research on Chronic Diseases, Beijing, 100084 China

**Dear Editor,**

Tumors evade immune surveillances, in part via negative regulatory pathways (also called checkpoints) that also regulate immune tolerance to autoimmunity (Thommen et al., 2018). Checkpoint inhibitor therapy, i.e., anti-CTLA-4 and anti-PD-1, has been approved to be an effective therapeutic approach in a variety of cancers (Mariathasan et al., 2018). However, only a subset of cancer patients shows durable responses (Callahan et al., 2016), urging for a broader investigation beyond PD-1 and CTLA-4. Renal cell carcinoma (RCC), the most common kidney cancer, was often considered as an immunogenic tumor based on high levels of T cell infiltration (Finke et al., 1992). However, the infiltrating T cells in RCC were reported to be characterized by a low amount of expanded T cell clonotypes (Sittig et al., 2013). Unexpectedly, the objective response rates to anti-PD-1 antibody were 18%–31% in PD-L1^+^ RCC patients vs. 9%–18% in PD-L1^−^ patients (Motzer et al., 2015; McDermott et al., 2016). Thus, there is an urgent need for investigation on immune evasion mechanisms in RCC, especially PD-1-independent ones.

We thus hypothesized that the low response rate to PD-1 blockade may be caused by co-expression of other checkpoint molecules in the immunosuppressive tumor microenvironment (TME). First, we analyzed the mRNA expression level of several checkpoint molecules in the B7 superfamily through GEPIA using data from TCGA and Oncoprint. We found there was no significant difference in *CD274* (encoding PD-L1) expression between RCC tumors and adjacent non-tumoral tissues (Fig. S1A), regardless of RCC types, clear cell RCC (ccRCC), chromophobe RCC (chRCC) or papillary RCC (pRCC). Notably, *C10orf54* (encoding VISTA) was significantly upregulated in tumors from patients with ccRCC and downregulated in chRCC tumors compared to adjacent non-tumoral tissues. *CD276* (encoding B7-H3) was highly expressed in tumors from patients with ccRCC as well as pRCC, whereas *VTCN1* (encoding B7S1) expression was significantly reduced in all RCC types compared to adjacent non-tumoral tissues. In addition, the expression levels of *CD276* and *C10orf54* were especially higher than *CD274* in ccRCC tumors (Fig. S1B). These data might underscore the low response rates to PD-1/PD-L1 inhibitors in ccRCC.

To evaluate the expression of the above checkpoint molecules at the protein level in ccRCC accounting for 75% of RCC, paired tumor and para-tumor tissues (2 cm away from tumors) were analyzed by immunofluorescence. The clinical and pathological characteristics of the patients were summarized in Table 1. Figures 1A and S2 show that VISTA was mostly expressed on CD45^+^ cells in para-tumors and tumors, consistent with published data that human VISTA is predominantly expressed in hematopoietic tissues and highly expressed within myeloid compartment (Lines et al., 2014; Ni and Dong, 2017b, a). Moreover, the expression level of VISTA in para-tumors was significantly lower than that in tumor sections (Fig. 1B), in line with the expression pattern of *VISTA* mRNA. In contrast, the expression levels of B7-H3 and B7S1 proteins were low in both para-tumors and tumors with no significant difference between the two samples, inconsistent with its mRNA expression pattern (Fig. 1A and 1B). PD-L1 was predominantly expressed by CD45^−^ cells (Figs. 1A and S2), and there was no significantly difference in PD-L1 expression between para-tumors and tumor tissues (Fig. 1B). To investigate whether ccRCC tumor cells express VISTA, sequential tumor sections were stained by anti-pan-cytokeratin and anti-VISTA, respectively. As shown in Figure 1C, pan-cytokeratin-expressing cells also showed VISTA expression, indicating that ccRCC tumor cells expressed VISTA, but at a relatively lower level.

**Figure 1:**
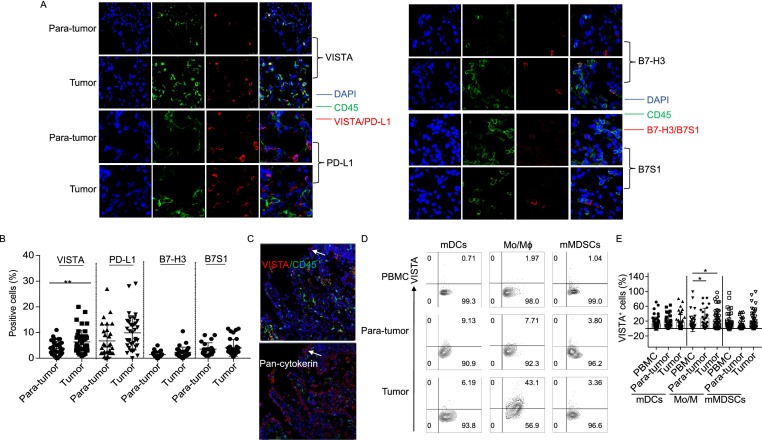
**VISTA protein is mainly expressed by intratumoral myeloid cells**. (A) Immunofluorescence analyses demonstrating the expression of VISTA, PD-L1, B7-H3 and B7S1 together with DAPI and CD45 in paired tumors and para-tumors. (B) Quantifications of VISTA, PD-L1, B7-H3 and B7S1 by immunofluorescence staining were shown (*n* = 47). **P < 0.01. (C) Immunofluorescence analyses demonstrating VISTA expression on tumor cells. (D and E) Representative figures and summarized data showing percentage of VISTA^+^ cells in mDCs, monocytes/macrophages, monocytic MDSCs from PBMC, para-tumors and tumors of ccRCC patients (*n* = 53). *P < 0.05

**Table 1:** Clinical and pathological characteristics of the ccRCC patients

Patient No.	Gender	Age	TNM stage	Tumor stage
P#1	male	59	T2N0M0	III
P#2	male	65	T1N0M0	II
P#3	male	48	T2N0M0	II
P#4	male	71	T2N0M0	II
P#5	male	56	T1N0M0	II
P#6	female	64	T2N1M0	III
P#7	male	52	T2N1M0	III
P#8	female	56	T1N0M0	II
P#9	female	59	T1N0M0	II
P#10	male	59	T1N0M0	II
P#11	male	48	T2N0M0	I
P#12	male	45	T1N0M0	II
P#15	male	58	T1N0M0	II
P#16	female	70	T1N0M0	II
P#17	male	49	T1N0M0	II
P#18	male	82	T2N0M0	II
P#19	female	35	T1N0M0	I
P#20	male	44	T1N0M0	II
P#23	male	51	T1N0M0	I
P#25	female	60	T2N0M0	II
P#26	male	61	T1N0M0	I
P#28	male	53	T2N0M1	III
P#29	male	64	T1N0M0	II
P#30	female	69	T1N0M0	II
P#31	male	74	T1N0M0	I
P#32	male	61	T1N0M0	I
P#34	female	56	T1N0MO	II
P#35	female	51	T2N0M1	III
P#37	female	76	T1N0M0	II
P#41	female	58	T2N0M1	III
P#43	female	77	T2N1M1	IV
P#44	male	67	T2N0M1	II
P#45	female	48	T3N0M0	II
P#46	male	52	T1N0M0	II
P#47	female	68	T1N0M0	II
P#48	male	29	T1N0M0	II
P#50	female	54	T1N0M0	II
P#51	male	46	T1N0M0	II
P#52	female	53	T1N0M0	III
P#53	male	64	T1N0M1	I
P#54	female	73	T1N0M0	II
P#55	male	70	T1N0M0	III
P#56	male	48	T1N0M0	II
P#57	female	70	T1N0M0	II
P#58	female	75	T1N0M0	II
P#59	female	57	T1N0M0	II
P#60	male	41	T3N0M1	I
P#62	male	48	T1N0M0	II
P#64	female	65	T1N0M0	I
P#65	male	54	T2N0M0	I
P#66	male	50	T3N0M0	III
P#68	male	65	T3N0M0	II
P#69	male	66	T1N0M0	I
P#EX-1	male	44	T1N0M0	II
P#EX-2	female	66	T1N0M0	I
P#EX-3	male	51	T1N0M0	I-II
P#EX-4	female	51	T1N0M0	II
P#EX-5	male	85	T2N1M0	II
P#EX-6	male	35	T1N0M0	II
P#EX-7	male	50	T1N0M0	I
P#EX-8	female	53	T2N0M1	II
P#EX-9	male	61	T2N0M1	II
P#EX-10	male	49	T1N0M0	I
P#EX-11	male	54	T1N0M0	II
P#EX-12	female	60	T2N0M0	II
P#EX-13	male	72	T2n0m0	II
P#EX-14	male	45	T1N0M0	II
P#EX-15	male	52	T1N0MO	I
P#EX-16	male	58	T2N0M1	III
P#EX-17	female	67	T2N0M0	II

Since VISTA is mainly expressed on tumor-infiltrated CD45^+^ cells, we next sought to identify which subsets of myeloid cells express VISTA. TILs from ccRCC patients were isolated following enzymatic digestion as our lab previously described (Xie et al., 2018). In addition, peripheral blood mononuclear cells (PBMCs) from the same patients were also used as controls. Different surface markers were employed to distinguish myeloid dendritic cells (mDCs, Lin^−^HLA-DR^hi^CD11C^+^CD123^−^), monocytes/macrophages (CD14^+^HLA-DR^hi^) and monocytic myeloid-derived suppressor cells (mMDSCs, CD14^+^HLA-DR^low/−^). As shown in Figure 1D–E, the percentages of VISTA-expressing macrophages in the tumors were significantly higher than in PBMCs and para-tumors. However, we did not find significant difference in VISTA expression on mDCs or MDSCs among PBMC, para-tumors and tumors. Consistent with the immunofluorescence results, PD-L1 was not expressed on myeloid cells in ccRCC patients (data not shown). Collectively, the above data not only confirmed the high prevalence of VISTA expression in ccRCC tumors at both mRNA and protein levels, but also revealed the distinct expression patterns of VISTA and PD-L1 in this cancer type.

VISTA is a negative immune checkpoint protein. VISTA expressed on APCs can suppress antigen-specific T cell activation during cognate interactions between APCs and T cells (Wang et al., 2011). Since CD14^+^HLA-DR^+^ macrophages in the ccRCC tumors expressed higher levels of VISTA, we sought to investigate the relevance of VISTA expression with infiltration and function of CD8^+^ TILs. To that end, ccRCC patients were divided into two groups (VISTA^hi^ and VISTA^low^) based on the average frequency of VISTA^+^ cells in all CD14^+^ myeloid cells (The cut-off value is 17.8%). Figure 2A and 2B show that significantly higher frequency of CD45^+^ TILs as well as CD3^+^CD8^+^ TILs was found in VISTA^low^ patients than VISTA^hi^ patients. In contrast, VISTA^low^ patients had significantly lower fractions of CD4^+^ TILs. These findings indicate that VISTA expression on intratumoral CD14^+^ myeloid cells was negatively associated with the degrees of CD8^+^ T cell infiltration. Moreover, CD8^+^ TILs in VISTA^low^ patients displayed higher co-expression of granzyme B and perforin than those in VISTA^hi^ patients (Fig. 2C and 2D). We also observed a significant increase in TNFα^+^CD8^+^ TILs in VISTA^low^ patients. However, these two groups expressed similar levels of IFNγ (Fig. 2C and 2D). These findings indicate that VISTA expression reversely correlates with the cytolytic function of CD8^+^ TILs, and more closely, with TNFα, but not IFNγ, expression. Due to very few NK TILs in some of the ccRCC patients, the correlation between VISTA expression and NK function could not be analyzed.

**Figure 2:**
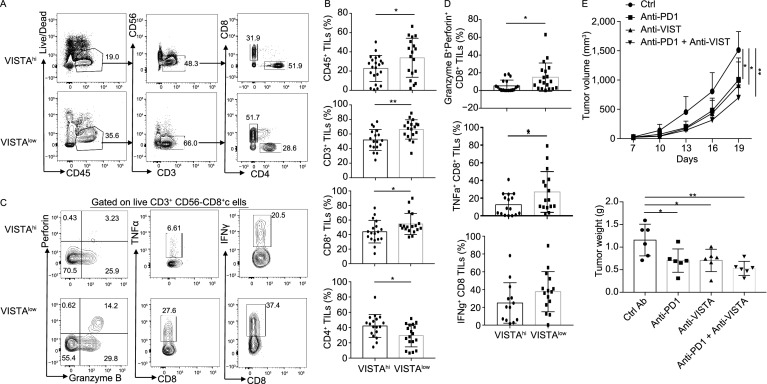
**Blockade of VISTA or PD-1 significantly inhibit kidney tumor growth**. (A and B) Representative figures and summarized data of infiltrated T cells in tumors of patients in VISTA^hi^ and VISTA^low^ groups. *P < 0.05, **P < 0.01. (C and D) Representative figures and summarized data displaying granzyme B, perforin, TNFα and IFNγ expression in CD8^+^ T cells in tumors. *P < 0.05, **P < 0.01. (E) Mean tumor volume and tumor weight of subcutaneous RENCA inoculation in mice treated with control antibodies, anti-VISTA, anti-PD-1, or anti-VISTA plus anti-PD-1 (*n* = 6). *P < 0.05, **P < 0.01

Having demonstrated that VISTA and PD-L1 may contribute to immune evasion in human ccRCC, we next sought to evaluate the efficacy of anti-VISTA alone or in combination with anti-PD-1 in a syngeneic mouse RCC model, RENCA. Murine VISTA is reported to be primarily expressed by hematopoietic cells and highly upregulated on APCs, but not on B cells, NK cells or granulocytes (Wang et al., 2011; Ni and Dong, 2017b). We found that the RENCA cell line exhibited strong PD-L1 but weak VISTA expression (Fig. S3A). We then investigated the expression patterns of VISTA and PD-L1 in this murine tumor model. Balb/c mice were subcutaneously inoculated with RENCA cells. On day 20, single cell suspensions of tumors were prepared and stained. VISTA was mainly expressed by CD45^+^ TILs, but was detected on very few intratumoral CD45^−^ cells. In line with human counterpart, mouse VISTA was highly expressed on CD11b^+^, F4/80^+^, and CD11c^+^ myeloid cells in the tumor (Fig. S3B). In contrast to VISTA, PD-L1 was mainly expressed by CD45^−^ cells and moderate PD-L1 expression was found on CD11b^+^ and F4/80^+^ myeloid cells (Fig. S3B).

To assess the function of VISTA, Balb/c mice were subcutaneously inoculated with RENCA cells and treated therapeutically with control Ig, anti-VISTA, anti-PD-1, or anti-VISTA plus anti-PD-1 antibodies on day 7, 10, 13, 16, and 19 via intraperitoneal injection. Figure 2E shows that either anti-VISTA or anti-PD-1 monotherapy significantly reduced tumor growth compared to control Ig treatment. Although combination of VISTA and PD-1 blockade also resulted in significant tumor reduction, the combined therapy did not elicit a synergistical effect on tumor growth compared with each monotherapy. Notably, we found very few CD3^+^ or CD8^+^ TILs, but abundant intratumoral myeloid cells (data not shown), consistent with a previous report on RENCA tumor model with disappear of all major T cell subpopulations in late tumors (Yu et al., 2018). Therefore, we could not analyze CD8^+^ TILs phenotypically and functionally. In contrast to clinical tumors that contained high percentage of T cells, the RENCA mouse model may not accurately recapitulate the human RCC tumor biology. A xenograft kidney tumor model should be utilized in future study to predict response and investigate the underlying molecular mechanism of this combined therapy.

This study is the first comprehensive phenotypic and functional analysis of VISTA in ccRCC tumors. Higher VISTA is detected in ccRCC tumors than that in non-tumoral tissues at both mRNA and protein levels. However, unlike PD-L1 that is mainly expressed by CD45^−^ cells, VISTA is highly expressed by intratumoral myeloid cells. ccRCC tumor cells also express VISTA at low levels. Notably, VISTA expression strongly correlated with poor CD8^+^ T cell responses and blockade of VISTA signaling significantly reduced the growth of murine RENCA RCC model. Taken together, these findings indicate that VISTA functions in suppressing tumor immunity and could serve as a target for immunotherapy in ccRCC.

## Electronic supplementary material

Below is the link to the electronic supplementary material.

Supplementary material 1 (PDF 1679 kb)
