# Supplementary material for: Analysis of VISTA expression and function in renal cell carcinoma highlights VISTA as a potential target for immunotherapy
Source: Protein Cell. 2019 Jun 24;10(11):840–5. doi: 10.1007/s13238-019-0642-z (PMC6834738; doi:10.1007/s13238-019-0642-z)
Supplement: Supplementary file 1 — Supplementary material 1 (PDF 1679 kb) [file 13238_2019_642_MOESM1_ESM.pdf]

Methods and materials

Patients and specimens

Fresh tumor samples, para-tumors, and matched blood without any preoperative therapies were obtained from patients undergoing nephrectomy or renal partial resection in the 8th Medical Center of Chinese PLA General Hospital and Peking University Third Hospital. Clinicopathological features of ccRCC patients are summarized in supplementary Table 1. All the procedures in this study were approved by the institutional review board at Tsinghua University and were performed in line with the institutional guidelines.

Isolation of peripheral blood mononuclear cells (PBMCs) and tissue/tumor-infiltrating leukocytes

Blood from ccRCC patients was drawn into heparinized tubes and centrifuged on Ficoll-Hypaque gradients (GE Healthcare Life Sciences). Fresh para-tumors and tumors from ccRCC patients were digested with 1mg/mL Collagenase A (Roche) supplemented with 10U/mL DNase I for 40 min at 37℃ prior to Ficoll-Hypaque gradient centrifugation. Isolation of tissue/tumor-infiltrating leukocytes was done according to the method described earlier (Lee et al., 2017).

Flow cytometry

The following fluorescent dye-conjugated anti-human antibodies were used for staining: anti-VISTA (730804) (R&D), anti-CD45 (HI30), anti-HLA-DR (L243), anti-CD11c (3.9), anti- B7H3 (DCN.70), anti-B7-S1 (MIH43), Streptavidin-BV421, anti-CD3 (OKT3), anti-CD56 (HCD56), anti-Perforin (dG9), anti-TNFα and anti-IFNγ (Biolegend); anti-PD-L1 (MIH1), anti-CD14 (MφP9), anti-CD123 (7G3), and anti-CD8 (SK1) (BD); anti-Granzyme B (GB11) (Invitrogen). Antibodies against mouse proteins were as follows: anti-CD45.1 (A20), anti-F4/80 (BM8), anti-Gr-1 (RB6-8C5), anti-

Ly6C (HK1.4), anti-MHC class II (I-A/I/E) (M5/114.15.2), anti-CD8a (53-6.7), anti-PD-1 (J43), anti-Granzyme B (NGZB), anti-Perforin (eBioscience), anti-Foxp3 (FJK-16 s), anti-IFN-γ (XMG1.2)(eBioscience); anti-CD11c (HL-3), anti-CD3e (145-2C11), anti-NK-1.1 (PK136), anti-CD4 (RM4-5), anti-TNFα (MP6-XT22)(BD); anti-CD11b (M1/70) and anti-VISTA (MH5A) (Biolegend); Mononuclear cell suspensions were stained with antibodies against surface molecules. For intracellular cytokine staining, cells were stimulated with PMA (50ng/mL, Sigma-Aldrich, MO) and ionomycin (500ng/mL, Sigma-Aldrich, MO) in the presence of Brefeldin A (Golgiplug, BD Bioscience) for 4 hours prior to staining with antibodies against surface proteins followed by fixation and permeabilization and staining with antibodies against intracellular antigens. Cells were acquired on an LSRFortessa (BD) flow cytometer, and data were analyzed using FlowJo X. Dead cells were excluded based on viability dye staining (Fixable viability dye eF506, eBioscience).

Immunofluorescence

All fresh samples were embedded in OCT and snapped in liquid nitrogen. 6μm-thick cryostat sections were cut and then incubated in 3% H_2O_2 for 15 min to destroy the activity of endogenous peroxidase. After blocking with 10% normal donkey serum, each slide was incubated with the diluted primary antibodies against PD-L1, VISTA, B7-H3, B7S1, CD45 or pan-cytokeratin in a humidified chamber at 4 ℃ overnight. The next day, incubate the slides with the second antibody AffiniPure F(ab')2 Fragment donkey anti-rabbit/mouse immunoglobulin antibody for 1hour at room temperature. Images were obtained by a fluorescence microscope (Zeiss LSM780). The quantification analysis was performed by ImageJ software.

Mice and RENCA tumor model

BALB/C mice were bred and kept under specific-pathogen free conditions in Animal

Facility of Tsinghua University. All animal protocols are approved by governmental and institutional guidelines for animal welfare.

1 × 10^6 RENCA cells were subcutaneously injected in a final volume of 100 μl PBS into the right flank of 8~12-week-old BALB/C mice, and tumor growth was monitored every 3 days. Tumor volume was calculated by the following formula: tumor volume =0.5×length×width^2. To test the therapeutic effect of combinational blockade of VISTA and PD-1, control antibodies, 100μg anti-VISTA (MH5A, Biolegend), 100μg anti-PD-1 (J43, Bioxcell) or 100μg anti-VISTA+100μg anti-PD-1 were intraperitoneally injected every 3 days starting from Day 7.

Statistical analysis

Statistical comparisons were determined with One-way ANOVA analysis followed by multiple comparisons or non-parametric Mann-Whitney test; Student's t test was performed for two-group analysis. *P* values less than 0.05 were considered to be statistically significant.

Supplemental Figure Legends

Figure S1. *VISTA* mRNA is highly expressed in ccRCC tumors.

(A) Overview of B7 family members in tumor tissue and adjacent non-tumoral tissue from different types of RCC. Data were shown on a log_2(TPM+1) scale using TCGA data. The major subtypes of kidney cancer were shown: ccRCC, chRCC and pRCC. TPM: transcripts per million. P value cutoff is 0.01. (B) Heatmap of the scaled log_2 fold change of B7/CD28 family genes expressed in RCC tumors. (C) Correlation of *C10orf54* expression with overall survival in ccRCC patients. ccRCC, clear cell RCC; chRCC, chromophobe RCC; pRCC, papillary RCC.

Figure S2. VISTA protein is mainly expressed by $CD45^+$ cells

Quantification of VISTA and PD-L1 on $CD45^{+/-}$ cells by immunofluorescence staining was shown (N=47). ***p<0.001.

Figure S3. Expressions of PD-L1 and VISTA

(A) Expression of VISTA and PD-L1 on RENCA cell line. (B) Expression of VISTA and PD-L1 in the tumor from RENCA-bearing mice (Day 20).

Lee, Y.H., Martin-Orozco, N., Zheng, P., Li, J., Zhang, P., Tan, H., Park, H.J., Jeong, M., Chang, S.H., Kim, B.S., *et al.* (2017). Inhibition of the B7-H3 immune checkpoint limits tumor growth by enhancing cytotoxic lymphocyte function. Cell Res 27, 1034-1045.

Figure S1

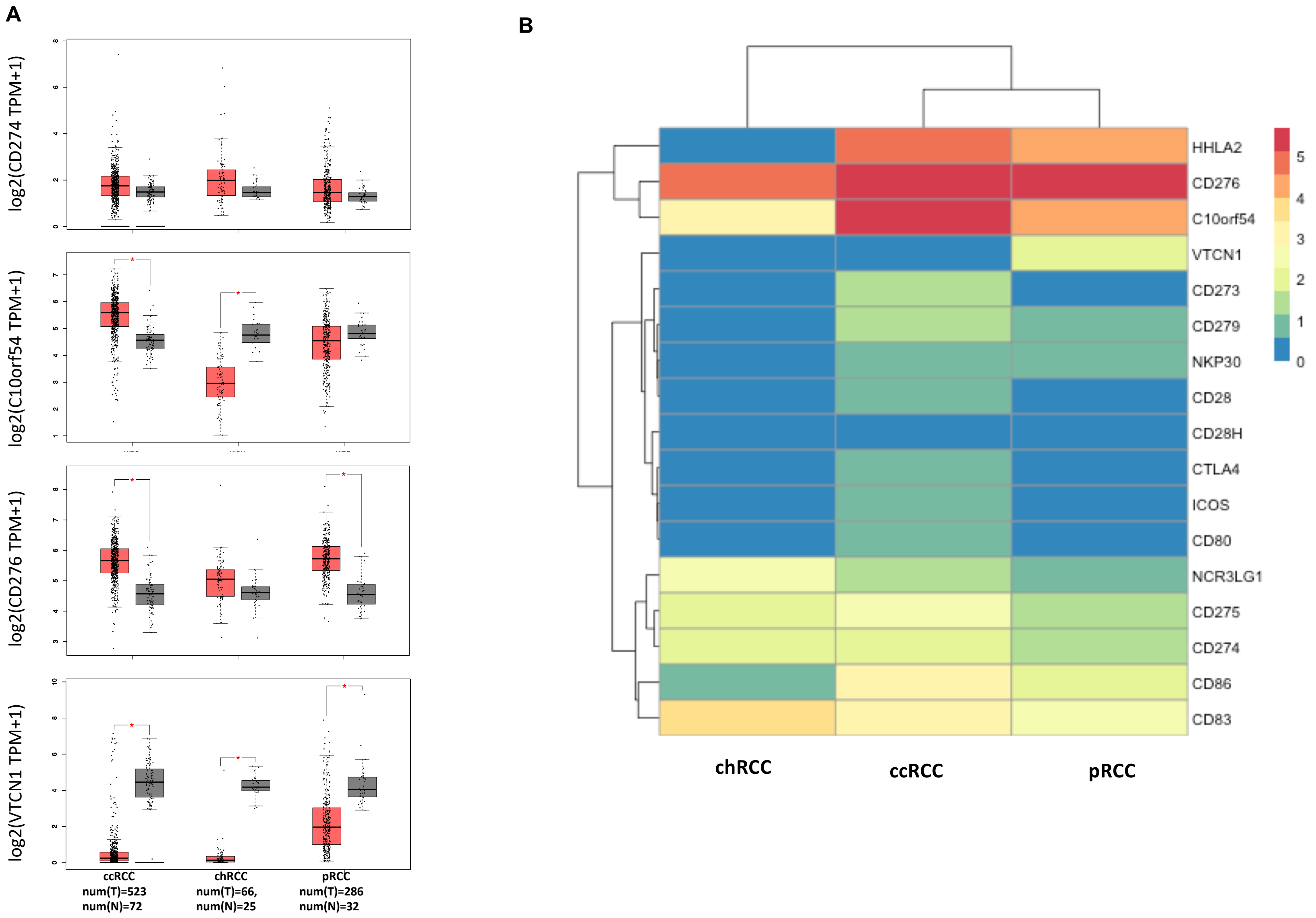
A
log2(CD274 TPM+1)
log2(C10orf54 TPM+1)
log2(CD276 TPM+1)
log2(VTCN1 TPM+1)
ccRCC
num(T)=523
num(N)=72
chRCC
num(T)=66,
num(N)=25
pRCC
num(T)=286
num(N)=32
B
HHLA2
CD276
C10orf54
VTCN1
CD273
CD279
NKP30
CD28
CD28H
CTLA4
ICOS
CD80
NCR3LG1
CD275
CD274
CD86
CD83
5
4
3
2
1
0
chRCC
ccRCC
pRCC

Figure S2

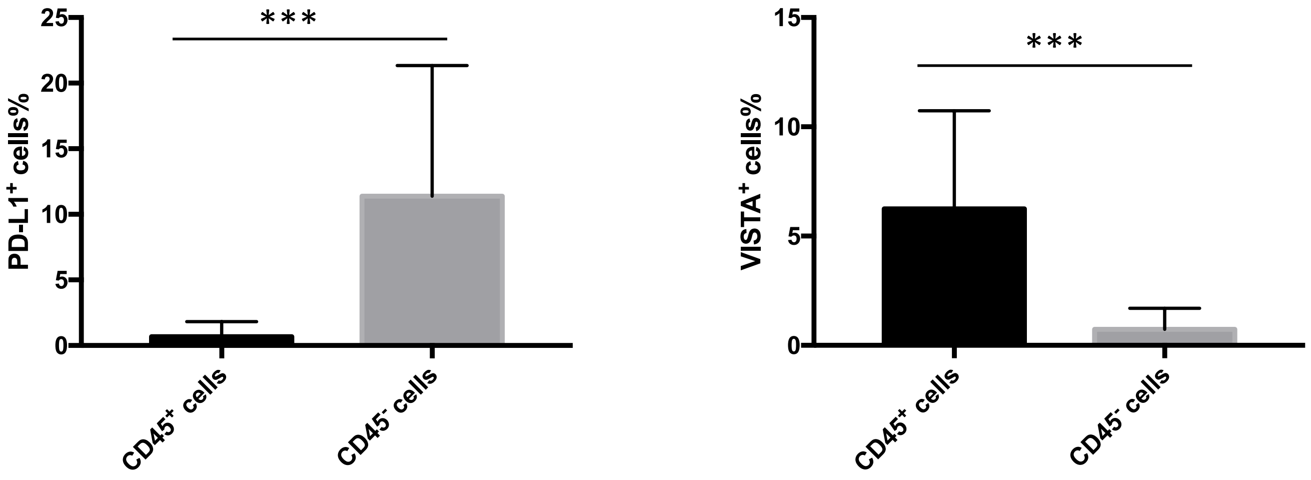

***
25
20
15
10
5
0
PD-L1+ cells%
CD45+ cells
CD45- cells
***
15
10
5
0
VISTA+ cells%
CD45+ cells
CD45- cells

Figure S3

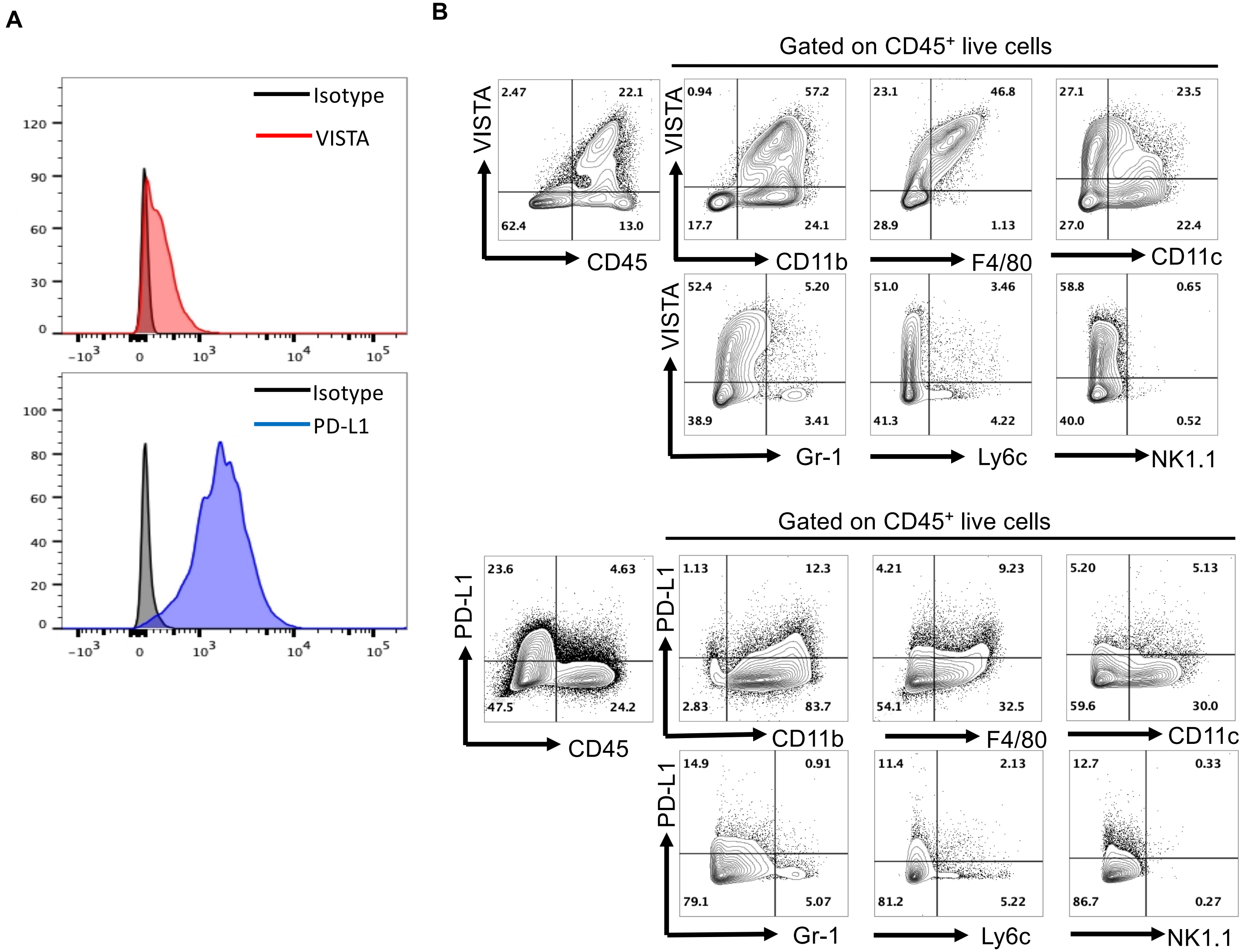
A
Isotype
VISTA
Isotype
PD-L1
B
Gated on CD45⁺ live cells
VISTA
CD45
CD11b
F4/80
CD11c
Gr-1
Ly6c
NK1.1
Gated on CD45⁺ live cells
PD-L1
CD45
CD11b
F4/80
CD11c
Gr-1
Ly6c
NK1.1
